# First-Principles Determination of Ultralow Thermal Conductivity of monolayer WSe_2_

**DOI:** 10.1038/srep15070

**Published:** 2015-10-14

**Authors:** Wu-Xing Zhou, Ke-Qiu Chen

**Affiliations:** 1 Department of Applied Physics, School of Physics and Electronics, Hunan University, Changsha 410082, China

## Abstract

By using first-principles calculations combined with the phonon Boltzmann transport equation, we systematically investigate the phonon transport of monolayer WSe2. Compared with other 2D materials, the monolayer WSe2 is found to have an ultralow thermal conductivity due to the ultralow Debye frequency and heavy atom mass. The room temperature thermal conductivity for a typical sample size of 1 μm is 3.935  W/m K, which is one order of magnitude lower than that of MoS_2_. And the room temperature thermal conductivity can be further decreased by about 95% in 10 nm sized samples. Moreover, we also find the ZA phonons have the dominant contribution to the thermal conductivity, and the relative contribution is almost 80% at room temperature, which is remarkably higher than that for monolayer MoS_2_. This is because the ZA phonons have longer lifetime than that of LA and TA phonons in monolayer WSe_2_.

As a family of two-dimensional materials beyond graphene, transition metal dichalcogenides (TMDCs) like MoS_2_ have gained a lot of attention due to many unique properties and potential applications^1^,^2^,^3^,^4^,^5^,^6^. Unlike zero-bandgap graphene, TMDCs possess a direct bandgap, which allows potential applications such as field effect transistors (FETs) and electroluminescent devices^7^,^8^. Very recently, the MoS_2_ is also regarded as a promising candidate for thermoelectric applications due to the large Seebeck coefficient and low thermal conductivity^9^,^10^,^11^,^12^,^13^,^14^. In general, the efficiency of a thermoelectric material to convert heat into electricity (and vice versa) is captured by a dimensionless figure of merit of the materials, defined as 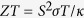, where 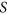 is the Seebeck coefficient, *σ* is the electronic conductivity, *T* is the absolute temperature, *κ* is the thermal conductivity including both the phononic contribution *κ*_*ph*_ and electronic contribution *κ*_*e*_. Therefore, low thermal conductivity is necessary to improve the thermoelectric energy conversion efficiency in thermoelectric application^15^,^16^,^17^,^18^.

WSe_2_, as another two-dimensional TMDCs, has much lower lattice thermal conductivity than MoS_2_^19^,^20^. An experimental measurement has reported that the cross-plane thermal conductivity of disordered WSe_2_ thin films is as low as 0.05 W/mK, which is the lowest thermal conductivity ever reported for a dense solid^19^, and the extremely low cross-plane conductivity is attributed to intensive phonon localization. Before long, the in-plane thermal conductivity of disordered WSe_2_ thin films was also reported by Shi and co-workers^20^. Their measurement results show that the in-plane thermal conductivity of the disordered layered WSe_2_ thin films is still about six times lower than that of compacted single-crystal platelets. These studies suggest that the WSe_2_ has great application potential in thermoelectric applications. In addition, recent experimental results are demonstrated WSe2 can be also used for making FETs and complementary inverters^21^,^22^,^23^, which has not yet been achieved on other TMDCs. In these applications, the thermal conductivity is a crucial parameter. Unfortunately, the systematical theoretical analysis on thermal transport properties of WSe_2_ is still in its infancy.

In addition, we know that there are three acoustic phonon modes in two-dimensional nanosheet, which are out-of-plane acoustic (ZA), transverse acoustic (TA) and longitudinal acoustic (LA) phonons, and the relative contribution of spectral phonons to thermal conductivity are crucial for understanding and modulating the thermal conductivity of nanostructures^24^,^25^. The relative contribution of different acoustic phonon modes to thermal conductivity of graphene and MoS_2_ has been widely studied^10^,^24^, however, this knowledge of WSe_2_ has not been reported.

Therefore, in the present work, we systematically investigate the influence of size, temperature and boundary roughness on the thermal conductivity, as well as the relative contribution of spectral phonons to thermal conductivity for monolayer well-ordered WSe_2_ by using first-principles calculations combined with the phonon Boltzmann transport equation (PBTE) with relaxation time approximation. The results show that the monolayer WSe_2_ is found to have an ultralow thermal conductivity, compared with other 2D materials. The room temperature thermal conductivity of monolayer WSe_2_ for a typical sample size of 1 μm is 3.935 W/m K, which is one order of magnitude lower than that of MoS_2_. And the room temperature thermal conductivity can be further decreased by about 95% in 10 nm sized samples. Moreover, we also found that the ZA phonons have the dominant contribution to the thermal conductivity in WSe_2_, the relative contribution to the thermal conductivity is almost 80% at room temperature, which is remarkably higher than that for monolayer MoS_2_. It is because the lifetime of ZA phonons is much longer than that of LA and TA phonons in monolayer WSe_2_.

## Results

**Figure 1:**
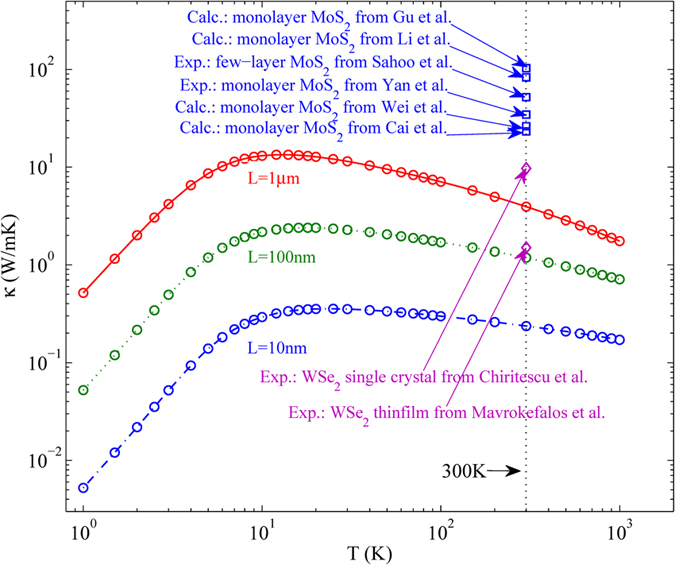
The temperature dependence of in-plane phonon thermal conductivity in monolayer WSe_2_ with different size. For purposes of comparison, the previous theoretical and experimental values of thermal conductivity in MoS_2_ and WSe_2_ are also presented.

**Figure 2:**
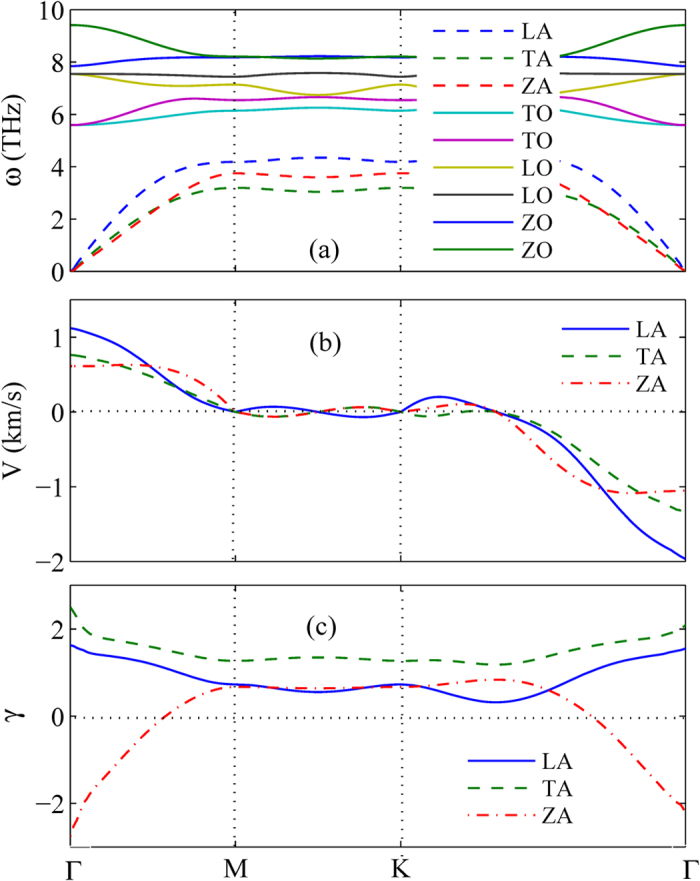
(**a**) Phonon dispersion relation, (**b**) phonon group velocity and (**c**) Grüneisen parameter of monolayer WSe_2_.

The temperature dependence of phonon thermal conductivity in monolayer WSe_2_ with different size is presented in Fig. 1. According to our calculation, the in-plane thermal conductivity of WSe_2_ is isotropic. For instance, the room thermal conductivity of WSe_2_ for a size of 1 μm along armchair and zigzag direction is 4.52 W/mK and 3.35 W/mK, respectively. Here, we take the average value of thermal conductivity along armchair and zigzag direction as the in-plane thermal conductivity. From Fig. 1, we can find that the room temperature thermal conductivity of monolayer WSe_2_ for a typical sample size of 1 μm is 3.935 W/m K, which is consistent with the previous experimental measurements, the diamonds taken from refs 20 and 21 as shown in Fig. 1, and the room temperature thermal conductivity can be further decreased to 0.23 W/mK in 10 nm sized samples. For purposes of comparison, we also give the previous theoretical and experimental values of thermal conductivity in MoS_2_ as shown in Fig. 1. For a sample size of 1 μm, the superior theoretical values of room temperature thermal conductivity of MoS_2_ are 83 W/mK and 103 W/mK, which calculated by solving phonon Boltzmann transport equation^10^,^26^. The inferior theoretical value of room temperature thermal conductivity in MoS_2_ is 23.3 W/mK calculated by using nonequilibrium Green’s function method^27^. The experimental values of room temperature thermal conductivity for monolayer and few-layer MoS_2_ are 34.5 W/mK and 52 W/mK, respectively^14^,^28^. Therefore, compared with monolayer MoS_2_, monolayer WSe_2_ has an ultralow thermal conductivity, which is one order of magnitude lower than that of MoS_2_. The ultralow thermal conductivity is due to the ultralow Debye frequency and heavy atom mass in monolayer WSe_2_. The influences of Debye frequency and atom mass to phonon thermal conductivity are also presented in previous research^29^. The relative atomic mass of MoS_2_ is 160.072, and the relative atomic mass of WSe_2_ is 341.76. So the atom mass of MoS_2_ is much heavier than that of WSe_2_. Moreover, from Fig. 2 (a), we can find that the Debye frequencies of TA, LA and ZA branch in WSe_2_ are 3.19 THz, 4.18 THz and 3.74 THz, and the Debye frequencies of TA, LA and ZA branch in MoS_2_ taken from ref. 13 are 4.80 THz, 7.20 THz and 5.70 THz, respectively. So the Debye frequencies of each branch in WSe_2_ are significantly lower than that in MoS_2_. Therefore, heavy atom mass and ultralow Debye frequency led to the ultralow thermal conductivity in monolayer WSe_2_.

In addition, from Fig. 1, we also find that the thermal conductivity of WSe_2_ increases with the increasing of temperature, and then decreases. This phenomenon can be understood from the two effects of temperature on thermal conductivity. On the one hand, with the increase of temperature, a growing number of phonons are excited to participate in thermal transport. This is the “positive” effect on thermal conductivity. On the other hand, the Umklapp phonon-phonon scattering is enhanced with the increase of temperature. This is the “negative” effect on thermal conductivity. Therefore, the influence of temperature on thermal conductivity depends on the competition of the two factors. At low temperatures, the Umklapp phonon-phonon scattering is very weak, the first effect is dominant, and so the thermal conductivity of WSe_2_ increases with the increasing temperature. At high temperatures, the Umklapp phonon-phonon scattering is dominant, so the thermal conductivity of WSe_2_ decreases with the increase of temperature.

**Figure 3:**
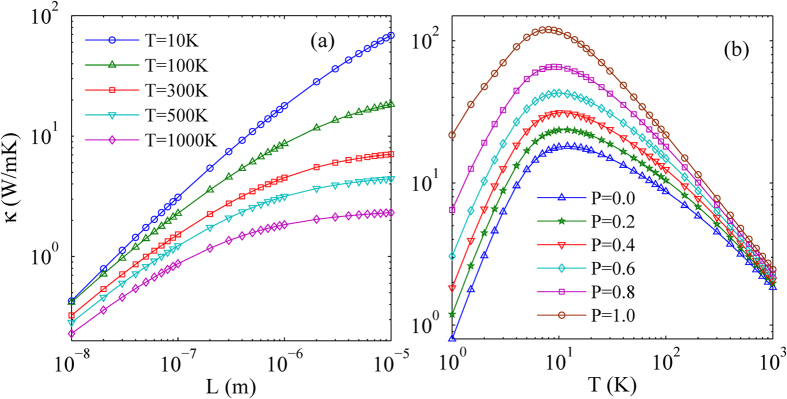
(**a**) The size dependence of in-plane thermal conductivity in monolayer WSe_2_ under different temperature. (**b**) The influence of boundary roughness on in-plane thermal conductivity for a typical sample size of 1 μm in monolayer WSe_2_.

The size dependence of thermal conductivity in monolayer WSe_2_ under different temperature is also researched as shown in Fig. 3(a). It is clearly shown that the thermal conductivity of monolayer WSe_2_ increases monotonically with the increasing of size under any temperature, however, the rate of increase is different under different temperature. Under low temperature such as 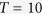 K, the increase of thermal conductivity is almost linear, that is to say, the rate of increase is almost constant. In contrast, under high temperature such as 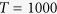 K, the increase rate of thermal conductivity decreases obviously with the increasing of size. This phenomenon can be well understood from the phonon scattering mechanism. Under low temperature, the linearly increasing behavior of thermal conductivity is a signature of the phonon-boundary scattering dominated thermal transport. It is because the Umklapp phonon-phonon scattering is very weak at low temperature, and the phonon-boundary scattering is dominant. When the sample size is less than the phonon mean free path, the phonon transport is ballistic, and the phonon mean free path increases linearly with the increasing of sample size leading to the linearly increasing behavior of thermal conductivity. However, with the increasing of temperature, the Umklapp phonon-phonon scattering is enhanced significantly leading to the decreasing of phonon mean free path, resulting in the decreasing of increase rate of thermal conductivity with the increasing of sample size. In fact, this behavior indicates a crossover from ballistic to ballistic-diffusive thermal transport. The similar phenomenon and interpretation also appears in the previous studies^30^,^31^,^32^. In short, the size dependence of thermal conductivity in monolayer WSe_2_ under different temperature depends on the competition of phonon-boundary scattering and Umklapp phonon-phonon scattering.

The influence of boundary roughness on thermal conductivity for a typical sample size of 1 μm is also researched, as shown in Fig. 3(b). As we know, the specular phonon scattering from a smooth interface (*p* = 1) is momentum-conserving, and does not add to thermal resistance. Only the diffuse phonon scattering from a rough interface (*p* < 1) adds to thermal resistance. It is because the diffuse phonon scattering changes the phonon momentum, limiting the phonon mean free path^33^. Therefore, we can find that the thermal conductivity increases monotonically with the increasing of the specularity parameter. In addition, as shown in Fig. 3(b), the increase rate of thermal conductivity at low temperature is far greater than that at high temperature. This is because the Umklapp phonon-phonon scattering is very weak at low temperature, and the phonon-boundary scattering is dominant. The phonon mean free path increases quickly with the increasing of specularity parameter. However, at high temperature, the Umklapp phonon-phonon scattering is enhanced, limiting the increase rate of phonon mean free path with the increasing of specularity parameter. Therefore, the increase rate of thermal conductivity at low temperature is far greater than that at high temperature with the increasing of specularity parameter.

**Figure 4:**
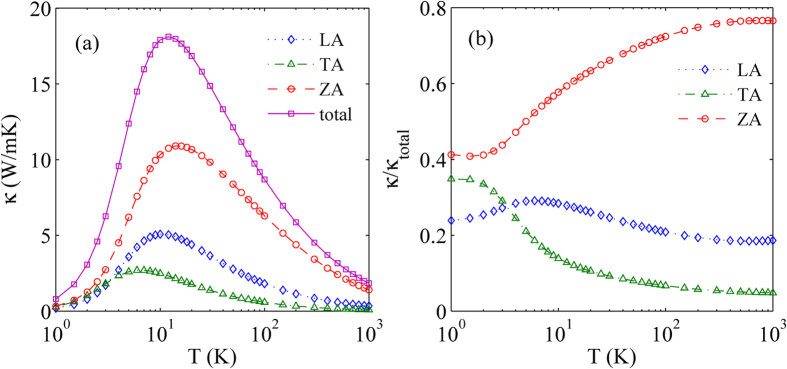
(**a**) The temperature dependence of in-plane thermal conductivity of LA, TA, and ZA branches with a size of 1 μm in monolayer WSe_2_. (**b**) The relative contribution of spectral phonons to in-plane thermal conductivity in monolayer WSe_2_.

The temperature dependence of thermal conductivity of spectral branch in a size of 1 μm is plotted in Fig. 4(a). It is clearly shown that the thermal conductivities of LA, TA, and ZA branches of WSe_2_ are all proportional to 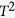 at low temperature. This is due to the linear dispersion of LA, TA and ZA branch near the Γ point as shown in Fig. 2(a)^34^. At high temperature, however, the thermal conductivities of LA, TA, and ZA branches approach a 1/T behavior, which is a signature of high temperature phonon-phonon scattering^34^. Moreover, we also give the relative contribution of spectral phonons to thermal conductivity in monolayer WSe_2_ as shown in Fig. 4(b). We can clearly find that the LA and TA phonons have relatively small contribution, and the ZA phonons have the dominant contribution to the thermal conductivity, and in a wide temperature range, the relative contribution of ZA phonons to the thermal conductivity increases remarkably with the increasing of temperature. At room temperature, the relative contribution of ZA phonons to the thermal conductivity is almost 80%, which is remarkably higher than that for monolayer MoS_2_^24^.

**Figure 5:**
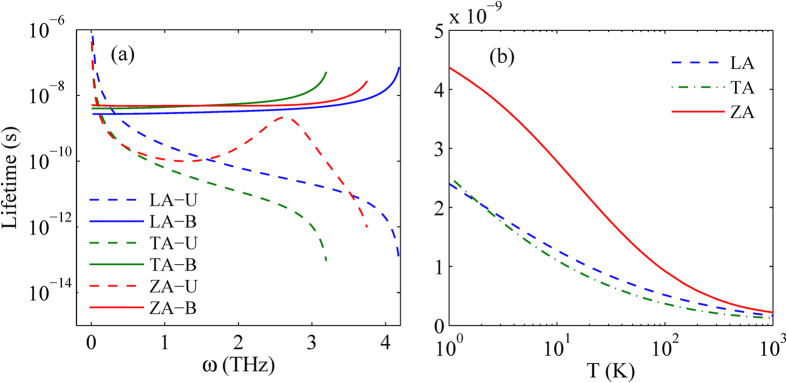
(**a**) The separate phonons lifetimes of LA, TA and ZA branches for Umklapp phonon-phonon scattering and phonon-boundary scattering with different frequencies in monolayer WSe_2_ with a size of 1 μm. (**b**) The averaged phonons lifetimes of LA, TA and ZA branches at different temperature in monolayer WSe_2_ with a size of 1 μm.

In order to understand this behavior, we calculate the separate phonons lifetimes of LA, TA and ZA branches for Umklapp phonon-phonon scattering and phonon-boundary scattering, as shown in Fig. 5(a). For the low frequency phonons, the phonon-boundary scattering is more significant than Umklapp phonon-phonon scattering, so the phonons lifetimes of LA, TA and ZA branches for Umklapp phonon-phonon scattering is longer than that for phonon-boundary scattering. With the increasing of phonon frequency, the Umklapp phonon-phonon scattering is enhanced, but the phonon-boundary scattering, which is dependent on the sample size and phonon group velocity, is almost constant below certain frequency and then increases. The increment is due to the phonon group velocity decreases obviously with the increasing of phonon frequency in the high frequency region, which can be seen from Fig. 2 (b). Therefore, for the high frequency phonons, the Umklapp phonon-phonon scattering is more significant than phonon-boundary scattering, leading to the shorter phonons lifetimes for Umklapp phonon-phonon scattering. In order to compare the separate phonons lifetimes of LA, TA and ZA branches intuitively, we calculate the averaged phonons lifetimes at different temperature in Fig. 5(b). The averaged phonons lifetimes of branch λ is derived as^24^ 
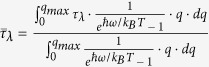
. From Fig. 5(b), we can clearly find that the phonons lifetimes of ZA branch is far greater than that of LA and TA branch over the whole temperature region. This result is in good agreement with the above conclusion that the ZA phonons have the dominant contribution to the thermal conductivity in monolayer WSe_2_.

## Discussion

In summary, by using first-principles calculations combined with the PBTE with relaxation time approximation, we systematically investigate the influence of size, temperature and boundary roughness on the thermal conductivity of monolayer WSe_2_. Compared with other 2D materials, the monolayer WSe_2_ is found to have an ultralow thermal conductivity. The room temperature thermal conductivity for a typical sample size of 1 μm is 3.935 W/m K, which is one order of magnitude lower than that of MoS_2_. And the room temperature thermal conductivity can be further decreased by about 95% in 10 nm sized samples. The ultralow thermal conductivity is due to the ultralow Debye frequency and heavy atom mass in monolayer WSe_2_. Moreover, we also investigate the relative contribution of spectral phonons to thermal conductivity in monolayer WSe_2_. The ZA phonons have the dominant contribution to the thermal conductivity, and the relative contribution is almost 80% at room temperature, which is remarkably higher than that for monolayer MoS_2_. This is because the ZA phonons have longer lifetime than that of LA and TA phonons in monolayer WSe_2_.

## Method

According to linearized PBTE within relaxation time approximation, the thermal conductivity in branch λ of monolayer WSe_2_ in the longitudinal direction of ribbon is derived as


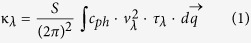


where λ = TA, LA and ZA^35^. Here, we only consider the contribution of three acoustic phonons to the thermal conductivity, because the contribution of optical phonons can be negligible due to the short phonon lifetime and small group velocity^10^. S is the area of the sample, *v*_*λ*_ is the phonon group velocity of λ branch along the longitudinal direction, *τ*_*λ*_ is the averaged phonon relaxation time (phonon lifetime) between successive scattering events of branch λ, 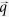 is the wave vector, and *c*_*ph*_ is the volumetric specific heat of each mode, which can be written as


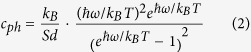


where *k*_*B*_ is the Boltzmann constant, 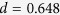 nm is the effective layer thickness of monolayer WSe_2_, which is assumed to be the interlayer spacing of bulk WSe_2_^26^. 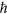 is the reduced Planck constant, and T is the absolute temperature. Using the phonon dispersion relation, the phonon group velocity of λ branch *v*_*λ*_ can be calculated as 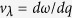, *ω* is the phonon frequency for branch λ at wave vector q.

The Matthiessen’s rule, which assumes that different scattering mechanisms are independent, is adopted to combine the effects of Umklapp phonon-phonon scattering 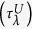 and phonon-boundary scattering 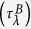, so the total phonon scattering rate 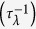, which is the inverse of the phonon lifetime, can be given as


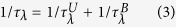


The 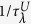, which is the Umklapp phonon-phonon scattering rate, can be written as^36^,^37^.


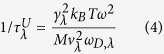


where 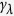 is the Grüneisen parameter, which characterizes the strength of the Umklapp phonon-phonon scattering process for branch λ. M is the mass of a WSe_2_ unit cell, and 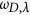 is the Debye frequency of branch λ. The scattering rate of phonon-boundary scattering can be given as^35^.


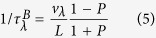


where L is sample size, and P is the specularity parameter, which is defined as a probability of specular scattering at the boundary. The specularity parameter ranges from 0 for a completely rough boundary to 1 for a perfectly smooth boundary^33^.

The properties such as phonon dispersion relation, phonon group velocity and Grüneisen parameter are calculated by using the PHONOPY code combined with Vienna ab-initio simulation Package (VASP) based on the density functional theory (DFT)^38^,^39^,^40^, as shown in Fig. 2(a–c). The project-augmented wave (PAW) potential and generalized-gradient approximation (GGA) exchange-correlation functional are adopted in our calculations^41^,^42^. The energy cutoff for the plane-wave expansion is set as 400 eV, and a Monkhorst-Pack k-mesh of 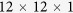 is used to sample the Brillouin zone, with the energy convergence threshold set as 10^−6 ^ eV. A 18.41 Å vacuum spacing is used to eliminate the interactions emerging from the employed periodic boundary conditions. The structures are fully relaxed until the maximal forces exerted on the atoms are no larger than 10^−6 ^ eV/Å. The optimized equilibrium lattice constant of monolayer WSe_2_ obtained in our calculation is 3.25 Å, which is in good agreement with previous experiment value of 3.27 Å^43^.

## Additional Information

**How to cite this article**: Zhou, W.-X. and Chen, K.-Q. First-Principles Determination of Ultralow Thermal Conductivity of monolayer WSe_2_. *Sci. Rep.* **5**, 15070; doi: 10.1038/srep15070 (2015).
